# Supplementary material for: The mechanosensitive ion channel TRAAK is localized to the mammalian node of Ranvier
Source: eLife. 2019 Nov 1;8:e50403. doi: 10.7554/eLife.50403 (PMC6824864; doi:10.7554/eLife.50403)
Supplement: Supplementary file 1. [file elife-50403-supp1.pdf]

Table 1 - Data collection and refinement statistics

	Anisotropic cutoff	Spherical cutoff
**Data Collection**		
Beamline	APS 24-IDE	
Wavelength (Å)	0.97915	
Number of crystals	2	
Space group	$P2_12_12_1$	
Cell dimensions		
a,b,c (Å)	72.45, 154.51, 202.85	
α=β=γ (°)	90	
Resolution (Å)	122.91-2.774	
shell where I/σI ~ 2 (Å)	3.141-2.774 (I/σI = 2.0)	3.755-3.571 (I/σI = 1.885)
R_{merge}	0.213 (1.589)	0.452 (34.263)
R_{meas}	0.221 (1.655)	0.469 (35.536)
R_{pim}	0.059 (0.457)	0.124 (9.357)
$CC_{1/2}$	(0.602)	(0.165)
I / σI	9.4 (2.0)	4.3 (0.1)
Completeness (%)	93.2 (80.2)	44.6 (7.3)
Multiplicity	14.1 (13.0)	14.5 (14.3)
**Refinement**		
No. of reflections used†	24830	
R_{work} (%)	24.8	
R_{free} (%)	28.6	
No. of total atoms	10250	
No. of K^+ ions	6	
Average B factor (Å2), all atoms	82.526	
Clashscore	7.3	
Molprobity score	1.77	
Ramachandran plot		
favored (%)	95.70	
allowed (%)	3.99	
disallowed (%)	0.31	
R.m.s. deviations		
Bond lengths (Å)	0.006	
Bond angles (°)	0.923	

*Values in parentheses are for the highest resolution shell.

†5% of these reflections were used to calculate Rfree
