# Supplementary material for: The mechanosensitive ion channel TRAAK is localized to the mammalian node of Ranvier
Source: eLife. 2019 Nov 1;8:e50403. doi: 10.7554/eLife.50403 (PMC6824864; doi:10.7554/eLife.50403)
Supplement: Supplementary file 2. [file elife-50403-supp2.pdf]

Target	Source animal	Supplier	Catalog number	Dilution	Conjugate
Primary antibodies					
Mus musculus TRAAK	Armenian hamster	this manuscript	na	20 ug/mL final	
TRAAK	Rabbit	this manuscript	na	1:500	
Caspr1	Rabbit	Abcam	34151	1:1000	
Caspr1	Mouse (IGG1)	Antibodies Incorporated	75-001	1:200	
K_V1.2	Mouse (IGG2B)	Antibodies Incorporated	75-008	1:200	
Pan Na_V	Mouse	Sigma	s8809	1:200-1:1000	
Ankyrin G	Mouse (IGG2A)	Antibodies Incorporated	75-146	1:125-1:200	
S100	Rabbit	Dako	Z0311	1:1000	
Keratin 8	Rat	Developmental Studies	TROMA-I-c	1:500	
Neurofilament H	Chicken	Aves	NFH	1:1000	
PSD95	Rabbit	Abcam	18258	1:1000	
GFAP	Chicken	Abcam	4674	1:1000	
IBA1	Rabbit	Wako	109-19741	1:500	
GFP	Chicken	Aves	GFP-1010	1:333	
Secondary Antibodies					
Arm. Hamster IgG	Goat	Jackson ImmunoResearch	127-545-160	1:500	Alexa 488
Arm. Hamster IgG	Goat	Life Technologies	A-21112	1:500	Alexa 568
Arm. Hamster IgG	Goat	Jackson ImmunoResearch	127-605-160	1:500	Alexa 647
Chicken IgY	Goat	Abcam	150173	1:500	Alexa 488
Chicken IgY	Goat	Abcam	175711	1:500	Alexa 568
Chicken IgY	Goat	Abcam	150175	1:500	Alexa 647
Rabbit IgG	Goat	Life Technologies	A-11034	1:500	Alexa 488
Rabbit IgG	Goat	Abcam	175471	1:500	Alexa 568
Rabbit IgG	Goat	Life Technologies	A-21244	1:500	Alexa 647
Mouse IgG	Goat	Abcam	150117	1:500	Alexa 488
Mouse IgG	Goat	Abcam	A150119	1:500	Alexa 647
Mouse IgG1	Goat	Life Technologies	A-21121	1:500	Alexa 488
Mouse IgG1	Goat	Life Technologies	A-21124	1:500	Alexa 568
Mouse IgG2a	Goat	Life Technologies	A-21131	1:500	Alexa 488
Mouse IgG2b	Goat	Life Technologies	A-21144	1:500	Alexa568
Rat IgG	Goat	Life Technologies	A-11006	1:500	Alexa 488
Rat IgG	Goat	Abcam	175710	1:500	Alexa568
Mouse IgG	Goat	Biotium	20018	1:500	CF-488A
Rabbit IgG	Goat	Biotium	20232	1:500	CF-555
